# Geriatric polytrauma patients should not be excluded from aggressive injury treatment based on age alone

**DOI:** 10.1007/s00068-020-01567-y

**Published:** 2020-12-15

**Authors:** Karlijn J. P. van Wessem, Luke P. H. Leenen

**Affiliations:** 1 Trauma Surgeon, Department of Trauma Surgery, University Medical Center Utrecht, Utrecht, The Netherlands; 2 Professor of Trauma Surgery, Department of Trauma Surgery, University Medical Center Utrecht, Utrecht, the Netherlands

**Keywords:** Geriatric polytrauma, Aggressive treatment, Withdrawal of life-sustaining therapy

## Abstract

**Purpose:**

Age in severely injured patients has been increasing for decades. Older age is associated with increasing mortality. However, morbidity and mortality could possibly be reduced when accurate and aggressive treatment is provided. This study investigated age-related morbidity and mortality in polytrauma including age-related decisions in initial injury management and withdrawal of life-sustaining therapy (WLST).

**Methods:**

A 6.5-year prospective cohort study included consecutive severely injured trauma patients admitted to a Level-1 Trauma Center ICU. Demographics, data on physiology, resuscitation, MODS/ARDS, and infectious complications were prospectively collected. Patients were divided into age subgroups (< 25, 25–49, 50–69, and ≥ 70 years) to make clinically relevant comparisons.

**Results:**

391 patients (70% males) were included with median ISS of 29 (22–36), 95% sustained blunt injuries. There was no difference in injury severity, resuscitation, urgent surgeries, nor in ventilator days, ICU-LOS, and H-LOS between age groups. Adjusted odds of MODS, ARDS and infectious complications were similar between age groups. 47% of patients ≥ 70 years died, compared to 10–16% in other age groups (*P* < 0.001). WLST increased with older age, contributing to more than half of deaths ≥ 70 years. TBI was the most common cause of death and decision for treatment withdrawal in all age groups.

**Conclusions:**

Patients ≥ 70 years had higher mortality risk even though injury severity and complication rates were similar to other age groups. WLST increased with age with the vast majority due to brain injury. More than half of patients ≥ 70 years survived suggesting geriatric polytrauma patients should not be excluded from aggressive injury treatment based on age alone.

**Supplementary Information:**

The online version contains supplementary material available at 10.1007/s00068-020-01567-y.

## Introduction

The last decades age of the general population increased globally with an increasingly active population. Consequently, an increasingly older polytrauma population has been observed. At the same time, advances in pre-hospital and in-hospital care have led to an increase in hospital admissions with an increasing proportion of geriatric patients [1, 2].

It has been widely demonstrated that there is an increased morbidity and mortality in older polytrauma patients [1, 3–7], although it is not completely clear whether this increased incidence is caused by a higher incidence of preexisting comorbidities, and/or a different reaction on physiological changes after trauma [1, 5, 8, 9]. Another important factor in geriatric patients is that mortality rates could also be influenced by (self-determined) limitation of therapy including withdrawal of life-sustaining therapy (WLST).

Even though care for the severely injured in general has improved with a decrease in mortality in the last decades, mortality rates in older patients are still high. Several authors have suggested to have specific guidelines for the management of severely injured elderly patients with on one hand special attention for more aggressive management initially, and on the other hand, more attention for withdrawal of care in case of medical futility [3, 10, 11].

To be able to compare research several researchers have tried to define ‘elderly’ by defining an age-cutoff point from which mortality increased significantly. These cut-off points ranged widely from as low as 47 years up to 70 years [12–14]. Since these reports originate from the early 2010s, and age is still increasing including in our trauma population, the aim of this prospective cohort study was to define a current age cut-off point for increasing mortality by investigating the relation of age on injury type and severity, physiology, complications (ARDS, MODS, infections) and mortality in a polytrauma population. Additionally, we investigated the role of age-related decisions in the initial management of injuries and withdrawal of life-sustaining therapy during hospital stay.

## Materials and methods

### Study setting

The study was conducted at an urban major (Level-1) trauma center. From November 2013, a 6.5-year prospective population-based cohort study was undertaken to investigate outcomes in severely injured patients admitted to the Intensive Care Unit (ICU) of the University Medical Center Utrecht. Detailed characteristics of the hospital and catchment area were previously described [15]. All consecutive severely injured trauma patients > 15 years of age who were admitted to ICU either directly from the emergency department (ED) or postoperatively after urgent surgery were included. Patients with isolated injury to the brain (Abbreviated Injury Score (AIS) head 3 or more and AIS 2 or less in other regions), asphyxiation, drowning and burns were excluded, because of the possible different physiologic response to severe trauma and a significantly different mortality and morbidity profile [16, 17].

### Data collection

All data were prospectively collected on arrival in ED and on a daily basis in ICU by the authors (KW, LL) and included patient demographics, Injury Severity Score (ISS), shock and resuscitation parameters. Both crystalloid and blood product (Packed Red Blood Cells (PRBC), Fresh Frozen Plasma (FFP) and Platelets (PLT)) administration was recorded in the first 24 h following admission. Denver Multiple Organ Failure (MOF) scores [18] and ARDS Berlin criteria [19] were registered daily up until 28 days or discharge from ICU. Denver MOF score was chosen over Sequential Organ Failure Assessment (SOFA) to avoid difficulties by including the Glasgow Coma Scale (GCS) in the organ failure score. GCS can be challenging to obtain in trauma patients in ICU because they are often sedated and intubated for extended periods. This could negatively influence the CNS organ failure score [17]. Further, infectious and thrombo-embolic complications were registered. Definitions and type of infectious complications that were registered have been previously described [20]. Further, Glasgow Outcome Score (GOS) that allows for objective assessment of the recovery of trauma patients in five categories was measured at discharge [21].

Additionally, patients were divided into four age groups to be able to make clinically relevant comparisons between young, middle-aged and elderly patients (< 25 years, 25–49 years, 50–69 years, and ≥ 70 years of age). Historically, patients over 65 years were typically regarded as being elderly. In recent decades life-expectancy in the developed countries has improved significantly with increased activity and mobility, and it was argued that 65 years might possibly be too young to be regarded as elderly these days. Therefore, age ≥ 70 years was defined as being elderly.

Primary outcome was the relation between different age groups and in-hospital morbidity and mortality. Secondary outcome was the relation between age-related decisions in the initial management of injuries and withdrawal of life-sustaining therapy (WLST) during hospital stay.

### Ethical approval

The local ethics committee approved this prospective observational study and waived consent (reference number WAG/mb/16/026,664).

### Statistical analysis

Data were analyzed using IBM SPSS Statistics, version 25.0 (Armonk, NY, USA). Graphs were prepared with GraphPad Prism version 8.3.0 (San Diego, CA, USA). Results are presented as median and interquartile range (IQR). Comparison of continuous variables was done using Kruskal–Wallis. Significant differences for categorical variables were calculated through Chi-Square test or Fisher’s exact test depending on the size of the groups. Variables with univariate statistical significance were included in a multinominal logistic regression analysis. These variables were analyzed to identify independent risk factors for the predefined age groups and presented as odds ratios and 95% confidence intervals. Statistical significance was defined as *P* < 0.05.

## Results

### Demographics whole studied population

391 patients (70% male) were included with a median age of 46 (IQR 28–62, range 80) years. Ninety-five percent of injuries were caused by a blunt mechanism, and median ISS was 29 (IQR 22–36) with 362 (93%) patients having an ISS ≥ 16. The most severe injuries were located in the brain (AIS head 3 (1–4) and chest (AIS chest 3 (2–4)). Eighty-two patients (21%) had a SBP ≤ 90 mmHg on arrival in ED. 249 (64%) patients had urgent surgery ≤ 24 h for various reasons (e.g. craniotomy, laparotomy, fracture fixation), and 119 (30%) sustained a pelvic fracture (Table 1). Patients received 4.6 (2.4–6.3) liters (L) of crystalloids ≤ 8 h and 7.4 (5.0–10.2) L ≤ 24 h. Further, they received 1 (0–4) unit of PRBC ≤ 8 h and 2 (0–5) units ≤ 24 h, 0 (0–4) units of FFP ≤ 8 h and 0 (0–5) units ≤ 24 h, 0 (0–1) units of PLT ≤ 8 h and 0 (0–1) units ≤ 24 h.

**Table 1:** Demographics and outcome

Demographics	Total population (*n* = 391)	Age < 25 (*n* = 76, 19%)	Age 25–49 (*n* = 142, 36%)	Age 50–69 (*n* = 101, 26%)	Age ≥ 70 (*n* = 72, 18%)	*P *value
Age (years)	46 (28–62)					
Male gender	272 (70)	56 (74)	105 (74)	74 (74)	37(51)	0.005*
Blunt MOI	373 (95)	73 (96)	134 (94)	95 (95)	71 (99)	0.50
Urgent laparotomy	95 (24)	24 (32)	41 (29)	21 (21)	11(15)	0.007*
Pelvic fracture	119 (30)	25 (33)	44 (31)	31 (31)	19 (26)	0.42
Urgent surgery ≤ 24 h	249 (64)	52 (68)	96 (68)	60 (59)	41 (57)	0.18
ISS	29 (22–36)	31 (25–36)	29 (22–38)	29 (22–37)	29 (20–35)	0.35
AIS head	3 (1–4)	3 (0–4)	3 (1–4)	3 (2–4)	3 (1–4)	0.16
AIS face	0 (0–2)	0 (0–2)	0 (0–2)	0 (0–2)	0 (0–1)	0.71
AIS chest	3 (2–4)	3 (2–4)	3 (2–4)	3 (2–3)	3 (2–4)	0.91
AIS abdomen	2 (0–3)	2 (0–3)	2 (0–3)	2 (0–2)	0 (0–2)	< 0.001*
AIS extr/pelvis	2 (0–3)	3 (2–3)	2 (0–3)	2 (0–3)	2 (0–3)	0.047*
AIS external	0 (0–1)	0 (0–1)	0 (0–1)	0 (0–1)	1 (0–1)	0.41
*Physiology and resuscitation*						
SBP_ED (mmHg)	120 (97–139)	121 (102–135)	120 (100–137)	120 (93–143)	116 (82–145)	0.63
SBP ≤ 90_ED	82 (21)	8 (11)	28 (20)	24 (24)	22 (31)	0.002*
Hb_ED (mmol/L)	8.0 (7.2–8.9)	8.0 (7.2–8.9)	8.2 (7.4–9.2)	8.2 (7.2–8.9)	7.2 (6.5–8.0)	0.005*
pH in ED	7.31 (7.25–7.36)	7.31 (7.25–7.37)	7.31 (7.24–7.35)	7.31 (7.27–7.36)	7.31 (7.21–7.38)	0.45
BD_ED (mmol/L)	3.0 (0.0–6.0)	2.0 (0.0–5.0)	3.0 (1.0–7.5)	2.0 (0.0–6.0)	3.0 (1.0–7.3)	0.20
PT_ED (sec)	14.9 (13.3–17.1)	16.0 (14.3–18.4)	14.5 (13.2–16.1)	14.3 (12.7–16.1)	15.1 (13.4–18.5)	0.21
SBP_ICU (mmHg)	119 (105–135)	120 (112–132)	119 (104–132)	119 (105–137)	116 (100–139)	0.82
Hb_ICU (mmol/L)	7.6 (6.8–8.3)	7.6 (7.0–8.4)	7.8 (7.0–8.3)	7.5 (6.8–8.5)	7.1 (6.2–7.8)	0.07
pH_ICU	7.33 (7.28–7.38)	7.34 (7.30–7.38)	7.33 (7.27–7.38)	7.34 (7.30–7.38)	7.33 (7.27–7.38)	0.71
BD_ICU (mmol/L)	4.0 (1.9–6.3)	3.5 (2.0–5.8)	4.2 (2.3–6.4)	3.5 (1.2–5.7)	4.4 (2.0–7.6)	0.79
UO_ICU (ml/hr)	150 (80–320)	175 (83–380)	150 (98–300)	150 (80–285)	133 (68–400)	0.07
PRBC ≥ 10 ≤ 24 h	39 (10)	9 (12)	14 (10)	10 (10)	6 (8)	0.89
*Outcome*						
Ventilator days	6 (2–11)	5 (2–10)	5 (2–10)	7 (2–11)	7 (2–12)	0.62
Vent free days	12 (4–19)	12 (6–18)	14 (7–19)	13 (4–19)	4 (0–16)	0.046*
ICU LOS (days)	7 (3–13)	6 (3–12)	6 (3–12)	8 (4–14)	9 (3–15)	0.22
H-LOS (days)	20 (11–31)	19 (10–30)	20 (13–31)	21 (13–32)	14 (7–31)	0.71
MODS	62 (16)	7 (9)	21 (15)	20 (20)	14 (19)	0.21
ARDS	16 (4)	3 (4)	6 (4)	5 (5)	2 (3)	0.82
Infectious complications	165 (42)	26 (34)	59 (42)	50 (50)	30 (42)	0.22
Thrombo-embolic complications	26 (7)	3 (4)	15 (11)	6 (6)	2 (3)	0.39
Mortality	74 (19)	12 (16)	14 (10)	14 (14)	34 (47)	< 0.001*

Data are expressed as absolute numbers (%) or medians (IQR)

*MOI *Mechanism of Injury, *ISS *Injury Severity Score, *AIS *Abbreviated Injury Scale, *ED *Emergency Department, *SBP *systolic blood pressure, *Hb *hemoglobin, *BD *Base Deficit, *PT *prothrombin time, *UO *urinary output, *PRBC *packed red blood cells, *vent free* *days* ventilator free days, *ICU *intensive care unit, *LOS *length of stay, *H-LOS *hospital length of stay, *MODS *multiple organ dysfunction syndrome, *ARDS *Adult Respiratory Distress Syndrome

*Statistically significant

Patients stayed 6 (2–11) days on the ventilator, 7 (3–13) days in ICU and 20 (11–31) days in the hospital. Sixty-two (16%) patients developed MODS, 16 (4%) ARDS, 165 (42%) developed infectious complications, and 26 (7%) thrombo-embolic complications (Table 1). Seventy-four (19%) patients died; the majority (70%) of them died of traumatic brain injury (TBI).

### Analysis of different age groups

**Fig. 1:**
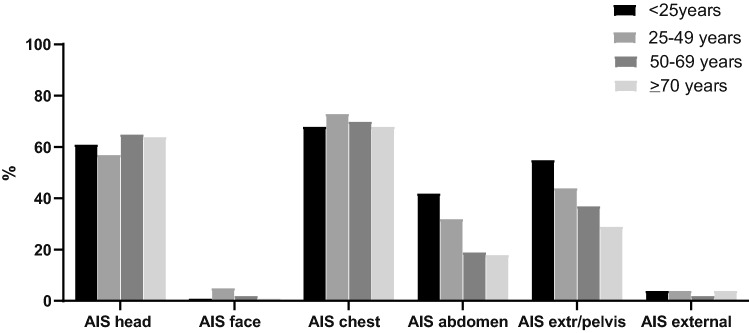
Abbreviated Injury Scale (AIS) ≥ 3 per injury region related to age

When analyzing different age groups, it was noted that with increasing age more females sustained severe injury. Further, the elderly had lower abbreviated injury score (AIS) of the abdomen and lower AIS extremities/pelvis even though ISS was similar between age groups (Table 1). There was no difference between age groups in a number of patients who underwent urgent surgery ≤ 24 h, although elderly underwent less frequently an urgent laparotomy. Figure 1 shows the percentage of AIS ≥ 3 per body region per age group. AIS head, face, chest and external were similar between age groups. AIS abdomen (*p* < 0.001) and AIS extremities/pelvis (*p* = 0.047) decreased with increasing age group.

**Fig. 2:**
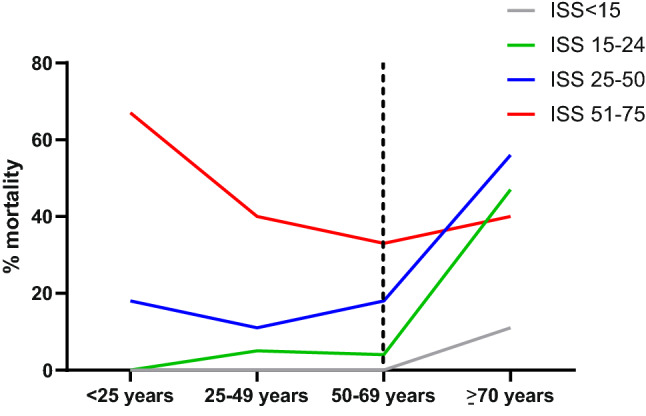
Mortality per age group related to the Injury Severity Score (ISS). The dotted line indicates the increase with age ≥ 70 years

Elderly had more often systolic blood pressure (SBP) ≤ 90 mmHg on arrival in ED and lower hemoglobin in ED (Table 1). There were no differences in crystalloid and blood product resuscitation between the age groups. Further, there was no difference in ventilator days, days in ICU nor in hospital, although the elderly had less ventilator-free days. No difference was found in incidence of MODS, ARDS, infectious complications nor thrombo-embolic complications between the age groups. In patients ≥ 70 years, 47% died compared to 10–16% in other age groups (*p* < 0.001, Table 1). There was no difference in mortality percentage between age groups < 25 years, 25–49 years and 50–69 years in ISS up to 50. Patients 70 and older died more often in ISS groups 15–24 and 25–50, but not in ISS < 15 or > 51. Caution should be exercised however in interpreting data in the highest ISS group since only 16 patients had ISS 51–75, and 7 (44%) of them died (Fig. 2).

**Fig. 3:**
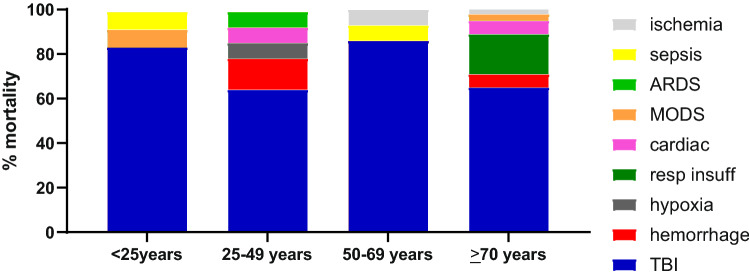
Cause of death per age group

The majority of patients in all age groups died of TBI. Elderly patients died more often of respiratory insufficiency compared to other age groups. In fact, all patients who died due to respiratory insufficiency were ≥ 70 years (Fig. 3).

In 36 patients (49%) who later deceased, withdrawal of life-sustaining therapy (WLST) was initiated because there was a negligible chance of recovery to an acceptable quality of life and treatment was therefore considered medically futile. This decision was more often made with increasing age; from 17% in deceased under < 25 years of age, to 36% in the age group 25–49 year, up to half the deceased patients aged 50–69, and 65% in the eldest patient group (*p* = 0.002, Table 2). TBI was the only cause for WLST in age groups up to 69 years. All 6 patients in whom respiratory insufficiency was the reason for WLST were ≥ 70 years; two of them developed hypercapnia due to respiratory insufficiency in high cervical spine injury (Table 2). The other 4 developed respiratory insufficiency on the ward where it was decided, in close harmony with patient (if possible) and family, against readmission to ICU for invasive ventilator support.

**Table 2:** Withdrawal of care in deceased patients

Mortality	Deceased population (*n* = 74)	Age < 25 (n = 12)	Age 25–49 (*n* = 14)	Age 50–69 (*n* = 14)	Age ≥ 70 (*n* = 34)	*P *value
WLST	36 (49)	2 (17)	5 (36)	7 (50)	22 (65)	0.002
Cause of death in WLST						
TBI	30 (83)	2 (100)	5 (100)	7 (100)	16 (73)	
Respiratory insufficiency*	6 (17)	0	0	0	6 (27)	0.21

Data are expressed as absolute numbers (%)

*WLST *withdrawal of life-sustaining therapy, *TBI *traumatic brain injury

*2 patients developed hypercapnia due to respiratory insufficiency due to high cervical spine injury

Thirty-four percent of surviving patients were discharged home with moderate to good recovery (GOS 4 and 5), and only 3% was discharged in a persistent vegetative state (GOS 2). The vast majority of surviving elderly patients were discharged to a rehabilitation center or nursing home (GOS 3), and were less likely to be discharged home directly from the hospital (*p* = 0.004, Table 3).

**Table 3:** Glasgow Outcome Score (GOS) at hospital discharge in surviving patients

	Age < 25 (*n* = 64)	Age 25–49 (*n* = 128)	Age 50–69 (*n* = 87)	Age ≥ 70 (*n* = 38)	Total (*n* = 317)
GOS 2 Persistent vegetative state	1 (2)	5 (4)	1 (1)	1(3)	8 (3)
GOS 3 Severe disability	35 (55)	74 (63)	61 (70)	33 (87)	203 (64)
GOS 4 Moderate disability	11 (17)	22 (19)	8 (9)	0	41 (13)
GOS 5 Good recovery	17 (27)	27 (23)	17 (20)	4 (11)	65 (21)

Data are expressed as absolute numbers (%)

**Fig. 4:**
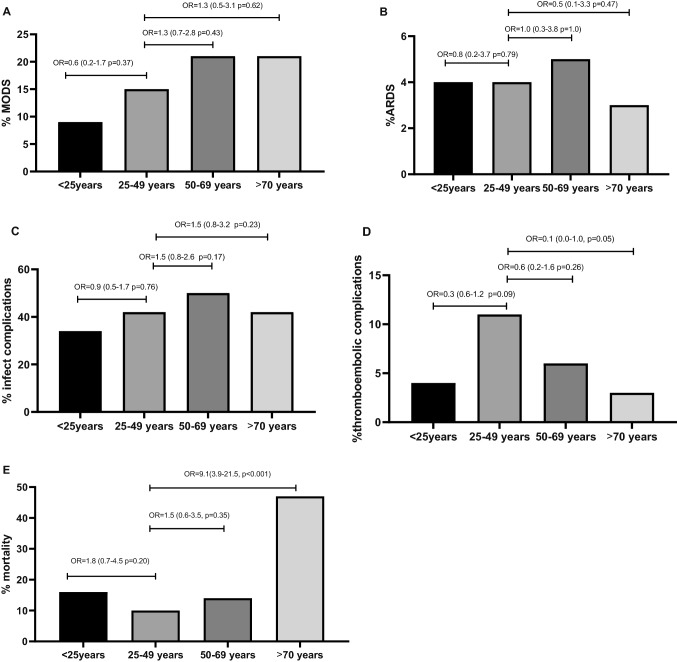
Adjusted odds ratio’s per age group for **a** MODS, **b** ARDS, **c** infectious complications, **d** thrombo-embolic complications, and **e** mortality

Multinominal logistic regression analysis was performed to identify possible independent outcome predictors for different age groups. Age group 25–49 years was used as a reference group. Gender, SBP ≤ 90 mmHg in ED, hemoglobin in ED and ICU, urgent laparotomy, and AIS abdomen were controlled for in the multinominal logistic regression analysis to avoid confounding. Adjusted odds ratios for MODS, ARDS, infectious and thrombo-embolic complications, and mortality were calculated and are shown in Fig. 4. The odds of MODS, ARDS and infectious complications were not statistically significant different between age groups. The odds of developing thrombo-embolic complications was 3 times lower patients < 25 years and 10 times lower in patients ≥ 70 years, although not statistically significant. Mortality increased ninefold in patients ≥ 70 years compared to the reference group (*p* < 0.001, Fig. 4).

## Discussion

In this prospective cohort study of polytrauma mortality was highest in elderly patients. Mortality increased with age ≥ 70 years independent of injury severity. In fact, patients over 70 years had 9 times higher chance of dying compared to patients between 25–49 years. In this study, the cut-off point from which mortality significantly increased was 70 years.

Interestingly, there was no difference in crystalloid and/or blood transfusion rates between the different age groups although elderly had more often SBP ≤ 90 mmHg and lower hemoglobin in ED, paradoxically less severe abdominal injuries and they underwent less often an urgent laparotomy. Since elderly suffer more often from hypertension than younger patients, SBP ≤ 90 mmHg in ED could be an expression of an even more deranged physiology and/or less functioning compensatory mechanisms than would be expected in younger patients. Additionally, Hatton et al. have previously demonstrated that the elderly might suffer from occult hypoperfusion even with normal vital signs. This is accompanied by worse outcomes compared with patients presenting with shock, because of either worsened underlying physiology or lack of timely detection and prompt treatment of this hypoperfusion [22]. This might partly explain worse outcomes in elderly with similar physiological parameters compared to their younger counterparts. Further, the observed lower hemoglobin in ED with similar acidosis compared to other age groups could possibly be explained by the fact that elderly have lower baseline hemoglobin due to various reasons (poor nutrition, anticoagulants, decreased bone marrow function).

There was no difference in complications such as MODS, ARDS, infections and thrombo-embolic complications between different age groups. The odds of thrombo-embolic complications in elderly was even 10 times lower than the reference group, this is possibly related to higher anticoagulant usage in the eldest age group. Similar MODS/ARDS rates between age groups are in contrast with various other studies, that showed an increase in MODS/ARDS and infections with increasing age [1, 5, 6]. A possible explanation for similar MODS/ARDS rates between age groups could be the low overall incidence of MODS and ARDS in the studied population. Another reason could be a decline in immune function seen in the elderly as postulated by Smith [23]. This could influence the ability to mount a normal immune response to major stress, so maybe elderly are at reduced risk of an immune modulated MODS or ARDS while more susceptible to post-injury infection due to reduced immune response to a new antigen. There is however little definitive evidence of this theory, and in the current study elderly did not develop more infectious complications than other age groups. It is tempting to argue that the elderly did not live long enough to develop complications, however, this is contradicted by the length of hospital admission that was comparable to other age groups.

In almost half the patients who later died withdrawal of life-sustaining therapy (WLST) was executed. WLST increased with increasing age and TBI was the only cause of WLST in patients up to 69 years. In patients ≥ 70 years respiratory insufficiency was another motive for WLST. In our institution age alone is not an exclusion for equal treatment in comparison with younger patients with adequate imaging and resuscitation, urgent surgery if necessary, and ICU admission. Our policy of no discrimination based on age alone is confirmed by current data with similarities between age groups regarding resuscitation volumes, urgent surgery rates, ventilator days, ICU and hospital length of stay. This is in contrast with a report from the German Trauma Registry that demonstrated that elderly polytrauma patients were more often treated with a ‘’wait and see approach’’ [11]. Others have shown similar data of less aggressive treatment in the elderly [6].

Sometimes chance of recovery to an acceptable quality of life is low and a no return to ICU policy could be advocated. This decision implicates that these elderly patients might expire from respiratory insufficiency if this would develop at a later stage during a hospital stay. This is in line with the current data showing that patients ≥ 70 years had highest mortality with least ventilator-free days and highest WLST (including due to respiratory insufficiency) suggesting that once it was decided that continuing care was futile, life-sustaining treatment was more often withdrawn in this age group. Additionally, there were few patients in a persistent vegetative state in all age groups. This is in agreement with our previous data on outcome in patients with moderate to severe isolated TBI [24]. Interestingly, a comparison study between Germany and the Netherlands in severe trauma patients revealed an almost none-existing rate of Dutch patients with persistent vegetative state compared to 4% in German patients [25]. We have previously speculated that these differences may be partly due to cultural differences [24]. Eighty-seven percent of surviving patients ≥ 70 years were discharged with GOS 3. In Glasgow outcome score there is no measured difference between patients who are discharged to a nursing home or to a rehabilitation facility (both GOS 3). Since these GOS data were calculated at discharge from hospital, an amelioration in recovery could be expected over time. In a previous study with moderate/severe TBI patients we have shown that patients at discharge from hospital improved over time with eventually more than half of the surviving patients with a good functional outcome [24]. Eleven percent of severely injured polytrauma patients over 70 years were discharged directly home from hospital suggesting it is worthwhile to have an aggressive approach in initial injury management.

One of the limitations of this study is that was conducted at a single institution in which the clinical treatment and research were conducted by the same clinicians. Another limitation is that no detailed past medical history data nor any data on GOS after discharge were collected. Further, in this study age ≥ 70 years was calculated as the cut-off point for increasing mortality. This cut-off point is somewhat artificial since it was based on age groups that were previously defined. In practice it is more likely there is a sliding scale for increasing mortality rather than an exact age cut-off point.

In conclusion, in this prospective cohort study polytrauma patients over 70 years had a nine times higher mortality risk even though injury severity and complication rates were similar to other age groups. Withdrawal of life-sustaining therapy contributed to more than half of deaths over 70 years with the vast majority due to brain injury. However, more than half of severely injured patients ≥ 70 years survived making it in our opinion worthwhile to have a similar initial aggressive approach as is a custom in younger patients. Age alone should not exclude elderly from initial aggressive treatment although restrictive treatment measurements later during hospital stay should be considered if it becomes apparent that chances of recovery to an acceptable quality of life are low.

## Supplementary Information

Below is the link to the electronic supplementary material.

Supplementary file1 (DOCX 30 KB)

## Data Availability

The dataset supporting the conclusions of this article are available upon reasonable request from the corresponding author.
